# Report of Signal Service at Baltimore

**Published:** 1880-01

**Authors:** Robert Seybath


### REPORT OF SIGNAL SERVICE OFFICE

AT BALTIMORE

FOR NOVEMBER, 1879.

| | |
|---|---|
| Mean Monthly Barometer, | 30.193 inches |
| Highest " | 30.595 " |
| Lowest " | 29.543 " |
| Mean Monthly Thermometer, | 46°.4 |
| Highest " | 78° |
| Lowest " | 20° |
| Mean Monthly Humidity, | 62.4 per cent. |
| Total Rainfall, | 1.30 inches |
| No. of Clear Days, | 10 |
| No. of Fair Days, | 16 |
| No. of Cloudy Days, | 4 |
| No. of Days on which Rain Fell, | 19 |
| Total Movement of Wind, | 4715 miles |
| Highest Velocity " | 30 " |

ROBERT SEYBATH.

